# Combined cisplatin and aurora inhibitor treatment increase neuroblastoma cell death but surviving cells overproduce BDNF

**DOI:** 10.1242/bio.016725

**Published:** 2016-06-02

**Authors:** Alessio Polacchini, Clara Albani, Gabriele Baj, Andrea Colliva, Patrizia Carpinelli, Enrico Tongiorgi

**Affiliations:** 1 University of Trieste, Department of Life Sciences, Trieste 34127, Italy; 2 Nerviano Medical Sciences, Nerviano, Milano 20014, Italy; 3 Department of Drug Discovery and Development, Istituto Italiano di Tecnologia, Genova 16163, Italy

**Keywords:** Neuroblastoma, Drug resistance, Neurotrophins, Cisplatin, Aurora kinase, PHA-680632

## Abstract

Drug-resistance to chemotherapics in aggressive neuroblastoma (NB) is characterized by enhanced cell survival mediated by TrkB and its ligand, brain-derived neurotrophic factor (BDNF); thus reduction in BDNF levels represent a promising strategy to overcome drug-resistance, but how chemotherapics regulate BDNF is unknown. Here, cisplatin treatment in SK-N-BE neuroblastoma upregulated multiple *BDNF* transcripts, except exons 5 and 8 variants. Cisplatin increased *BDNF* mRNA and protein, and enhanced translation of a firefly reporter gene flanked by BDNF 5′UTR exons 1, 2c, 4 or 6 and 3′UTR-long. To block BDNF translation we focused on aurora kinases inhibitors which are proposed as new chemotherapeutics. NB cell survival after 24 h treatment was 43% with cisplatin, and 22% by cisplatin+aurora kinase inhibitor PHA-680632, while the aurora kinases inhibitor alone was less effective; however the combined treatment induced a paradoxical increase of BDNF in surviving cells with strong translational activation of exon6-3′UTR-long transcript, while translation of *BDNF* transcripts 1, 2C and 4 was suppressed. In conclusion, combined cisplatin and aurora kinase inhibitor treatment increases cell death, but induces BDNF overproduction in surviving cells through an aurora kinase-independent mechanism.

## Abstract (precis)

**Summary:** Cisplatin increases endogenous BDNF in MYCN-expanded neuroblastoma cells. Additional treatment with aurora kinase inhibitor PHA-680632 increases cell death but surviving cells overproduce BDNF, mainly by increased translation of exon 6.

## INTRODUCTION

Neuroblastoma (NB) is a solid tumour derived from the sympatho-adrenal lineage of the neural crest cells and is the most common paediatric tumour accounting for 15% of cancer-related death in children (Nakamura et al., 2014). NBs can have either a favourable or an unfavourable prognosis depending on intrinsic features including the expression of neurotrophic factors of the neurotrophin family and their receptors, which are involved in cell survival and differentiation during development (Skaper, 2012; Brodeur et al., 2009). In particular, favourable NBs express TrkA (the receptor for nerve-growth factor) while unfavourable NBs express TrkB and its ligand, brain-derived neurotrophic factor (BDNF), and amplification of the oncogene *MYCN* (Brodeur et al., 2009; Buhagiar and Ayers, 2015). There is consolidated evidence that increased expression of BDNF and its receptor TrkB, working in an autocrine/paracrine way, is able to confer resistance to chemotherapeutics, leading NBs to display enhanced cell survival and aggressiveness (Nakamura et al., 2014; Nakagawara et al., 1993). Indeed, overexpression of TrkB blocks drug-induced cell death in a dose-dependent manner (Jaboin et al., 2002). Similar effects can also be obtained by BDNF treatment, while drug susceptibility can be restored after TrkB inhibition using K252a (Jaboin et al., 2003, 2002) or blocking BDNF availability using anti-BDNF neutralizing antibodies (Ho et al., 2002; Feng et al., 2001). The mechanism of BDNF pathway-mediated resistance to the most commonly used anti-tumour drugs in NB (cisplatin, vinblastine, etoposide and doxorubicin) has been described. BDNF/TrkB contributes to drug resistance and cell survival by increasing phosphorylation of AKT via the phosphatidylinositol 3′-kinase (PI3K)/AKT pathway (Ho et al., 2002), and the inhibition of PI3K restores cisplatin-induced cytotoxicity (Jaboin et al., 2003).

**Fig. 1.**
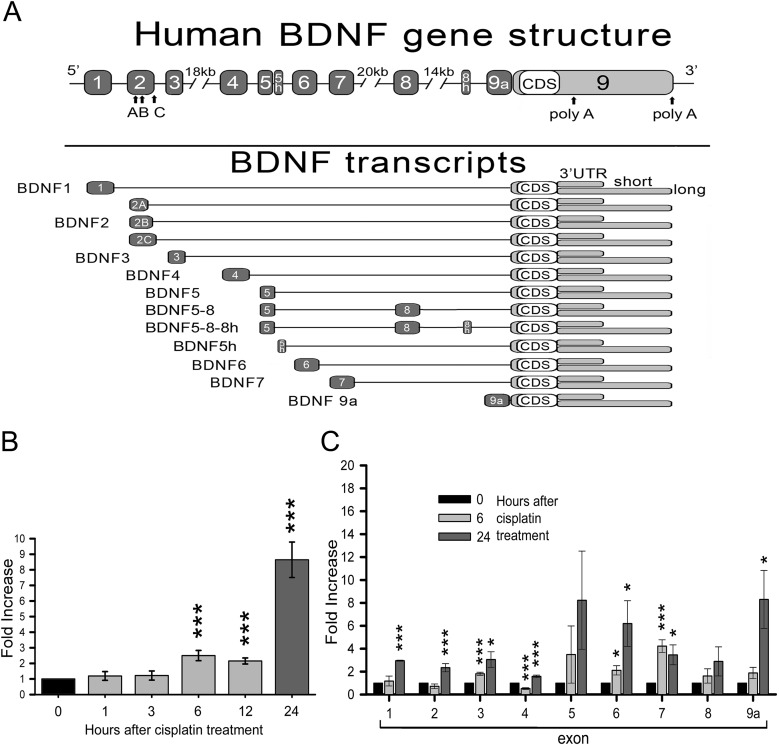
**Increment in BDNF transcripts after cisplatin treatment.** (A) BDNF human gene structure according to Pruunsild et al. (2007). We represented only isoforms that were relevant for this study. (B) Total BDNF CDS expression in SK-N-BE cells (differentiated 4.5 DIV) and treated in serum free medium (SFM)+Cisplatin (5 µg/ml). RNAs were extracted at different time points after cisplatin treatment: 0 (untreated control), 1, 3, 6, 12, 24 (*N*=3). (C) Modulation of single BDNF mRNA isoforms (exons are given in the x-axis) at 6 and 24 h of cisplatin treatment, normalized to the untreated control (0) (*N*≥4). B and C data were collected through real time PCR with SYBR^®^ Green Technology and data are given as mean±s.e.m. (*P<0.05, **P<0.01, ***P<0.001; one-way ANOVA).

BDNF is a secreted, small dimeric protein which is produced from a large number of transcripts generated by alternative splicing of eight upstream exons encoding the 5′ untranslated region (UTR), and a common downstream exon 9 containing a unique coding region and a short or long 3′UTR region (Fig. 1A) (Aid et al., 2007; Pruunsild et al., 2007). In a previous study, using siRNAs targeted to each individual *BDNF* transcripts, we demonstrated that siRNAs against either the coding sequence (exon 9) or the isoforms 4, 6, 9a that are located in the second exon cluster were able to decrease the survival of SK-N-BE neuroblastoma cells (a model carrying *MYCN* amplification) following treatment with cisplatin (Baj and Tongiorgi, 2009). These results suggested that *BDNF* transcript-specific silencing could aid in increasing the efficacy of treatments for drug-resistant NBs.

Inhibitors of aurora kinases have recently been proposed as a novel class of antitumoral chemotherapeutics to target drug-resistant NBs (Maris, 2009; Michaelis et al., 2014). The aurora kinase family comprises three serine/threonine kinases (AURKA, AURKB, and AURKC) involved in centrosome function and spindle organization during mitosis, and in the regulation of the cell cycle (Fu et al., 2007). These kinases have gained interest as drug targets since they act as oncogenic drivers in many human cancers (Maris, 2009). Aurora kinases expression and amplification, especially AURKA, are negative prognostic markers indicating high-risk disease in drug-resistant neuroblastoma cells (Michaelis et al., 2014). In addition, AURKA stabilizes N-Myc protein which is normally degraded following low levels of PI3K activity, thus promoting mitosis exit (Otto et al., 2009). Aurora kinases also regulate translation through the phosphorylation of cytoplasmic polyadenylation element binding protein (CPEB), which binds to cytoplasmic polyadenylation element (CPE) on mRNA transcripts (Groisman et al., 2006; Otto et al., 2009). Importantly, this is a conserved mechanism being described from *Xenopus* oocytes to human neurons where, in addition to translational regulation, it is also involved in dendritic mRNA trafficking (Huang et al., 2002). It is noteworthy that all *BDNF* transcripts include a CPE element in the 3′UTR (Oe and Yoneda, 2010; Vicario et al., 2015). In this study, we tested the hypothesis that cisplatin might induce drug resistance in one aggressive *MYCN*-amplified neuroblastoma by affecting the regulation of endogenous BDNF levels. Secondly, we attempted to increase cell mortality by combining cisplatin with the aurora kinase inhibitor PHA-680632, which we used to prevent BDNF translation. Finally, we evaluated the BDNF protein production in the neuroblastoma cells which survived the combined treatment.

## RESULTS

### Cisplatin treatment increases BDNF in differentiated SK-N-BE neuroblastoma cells

We previously demonstrated that SK-N-BE neuroblastoma cells differentiated with 9-cis retinoic acid show higher production of BDNF and its receptor, TrkB. Differentiated cells resulted to be more resistant to cisplatin-induced death with respect to undifferentiated cells, and silencing of BDNF attenuated this effect (Baj and Tongiorgi, 2009). Given these observations, we investigated whether cisplatin is able to modulate BDNF expression. SK-N-BE cells were differentiated for 4.5 days with 9-cis retinoic acid followed by cisplatin treatment (5 µg/ml) in serum-free medium for 1, 3, 6, 12 and 24 h. After RNA extraction and retro-transcription, total *BDNF* mRNA was quantified through real time PCR (*N*=3 independent cultures). We found a significant increase (2.5-fold, ***P<0.001) in BDNF coding sequence mRNA expression after 6 h, which was stable up to 12 h and was more pronounced (8.6-fold, ***P<0.001) after 24 h of treatment (Fig. 1B). BDNF has a complex gene structure (see Fig. 1A) characterised by different 5′-untranslated regions (UTR), each encoded by a different exon spliced to a common downstream exon containing the coding sequence (CDS) and the 3′UTR that exists in two forms, one short and one long, due to the presence of two polyadenylation sites. The different *BDNF* splice variants are transcribed independently, and in humans give rise to a total of 34 possible transcripts (Pruunsild et al., 2007, 2011; Aid et al., 2007). We found that the increase in BDNF expression induced by cisplatin was not limited to the coding sequence but involved almost all transcript variants (1, 2, 3, 4, 6, 7 and 9a), especially after 24 h of treatment. Exons 5 and 8 variants were only slightly increased, although not significantly. Expression of exons 3, 6 and 7 was increased already after 6 h of cisplatin treatment, while we observed a transient decrease in exon 4 expression at this time point (Fig. 1C).

**Fig. 2.**
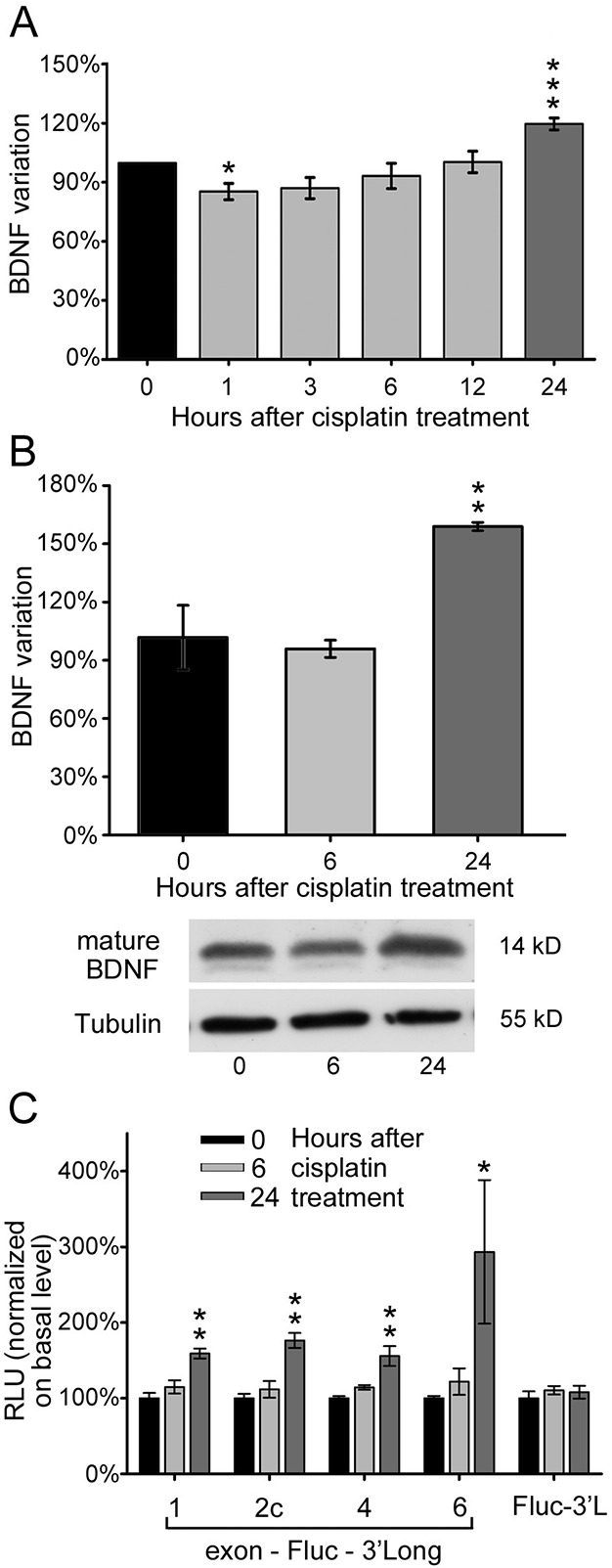
**Cisplatin increases both BDNF protein production and mRNA translational efficiency.** (A) ELISA detection of total BDNF protein level in 4.5 DIV differentiated SK-N-BE cells after cisplatin treatment, at different time points: untreated control (0), 1, 3, 6, 12 and 24 h (*N*=4, each in duplicate). (B) Evaluation of mature BDNF protein production through densitometric analysis of western blot, in the same conditions as described in panel A, but limited to time point treatment of 6 and 24 h; BDNF signal is normalized to tubulin and given as percentage of variation with respect to control. Lower panel: representative western blot of mature BDNF and tubulin (*N*=3). (C) Translational efficiency of different BDNF isoforms under cisplatin cytotoxic stress, at 6 and 24 h time points. The isoforms tested are as follows: exon1-Fluc-3′UTR long (Ex1-F-3′L), exon2c-Fluc-3′UTR long (Ex2c-F-3′L), exon4-Fluc-3′UTR long (Ex4-F-3′L) and exon6-Fluc-3′UTR long (Ex6-F-3′L). Values from each isoform are normalized for transfection efficiency over a control vector (Rluc) and given as fold increase on basal condition (0); *N*=3, each in duplicate; RLU: relative luciferase unit. In all panels, data are represented as mean±s.e.m. *P<0.05, **P<0.01, ***P<0.001; one-way ANOVA versus untreated control.

We then verified if the increased expression of *BDNF* transcripts corresponded to a change in BDNF protein production upon the same treatments. Using a sandwich ELISA normalized by loading the same total protein amount, we observed a slight but significant decrease in BDNF protein levels at 1 h of cisplatin treatment and a return to control levels after 12 h. Treatment for 24 h enhanced BDNF protein levels by around 20% above the basal level (untreated control; ***P<0.001, one-way ANOVA; see Fig. 2A). Similar results were obtained also by western blot using a semi-quantitative densitometric analysis of the mature BDNF band signals normalized to tubulin bands. Indeed, there was no significant variation in mature BDNF levels after 6 h, but 24 h of treatment induced about 30% increase in mature BDNF protein (**P=0.007, one-way ANOVA; Fig. 2B).

Since the different BDNF transcripts are upregulated by cisplatin treatment, we investigated which of them is more efficiently translated, hence contributing mostly to BDNF protein production. In a previous study from our laboratory, we developed an *in vitro* translation assay by which we demonstrated that different *BDNF* transcripts display a different efficiency of translation, basally and after stimulation, and this ability is influenced by the presence of both 5′ and 3′UTRs (Vaghi et al., 2014). Accordingly, we transfected SK-N-BE cells with a construct holding a firefly luciferase (Fluc) reporter gene flanked by different 5′UTR (exons 1, 2c, 4 and 6, alternatively) and the 3′UTR long. Exons 1, 2, 4 and 6 are the most abundant *BDNF* transcripts accounting together for 96% *BDNF* mRNA in the brain (Baj et al., 2013). In order to normalize for differences in transfection efficiency we co-transfected the same cells with a control vector bearing a second reporter, namely *Renilla* luciferase (Rluc). After treatments, Fluc bioluminescence activity was recorded and double-normalized over the Rluc activity and the untreated condition within the same experiment. After 24 h of cisplatin treatment, we observed that exons 1, 2c, 4 transcripts showed a significant increase in translation (**P<0.010; Fig. 2C); while exon 6 translation although significantly increased (*P=0.020) displayed a high variability, possibly due to the fact that exon 6 is translationally repressed in basal conditions. Of note, the 3′UTR long alone does not substantially contribute to the efficiency of translation of BDNF following cisplatin treatment, in analogy with other drugs tested in a previous study (Vaghi et al., 2014). These results indicate that the production of BDNF is increased in the subpopulation of surviving cells.

### An aurora kinases inhibitor enhances the cisplatin cytotoxic effect

**Fig. 3.**
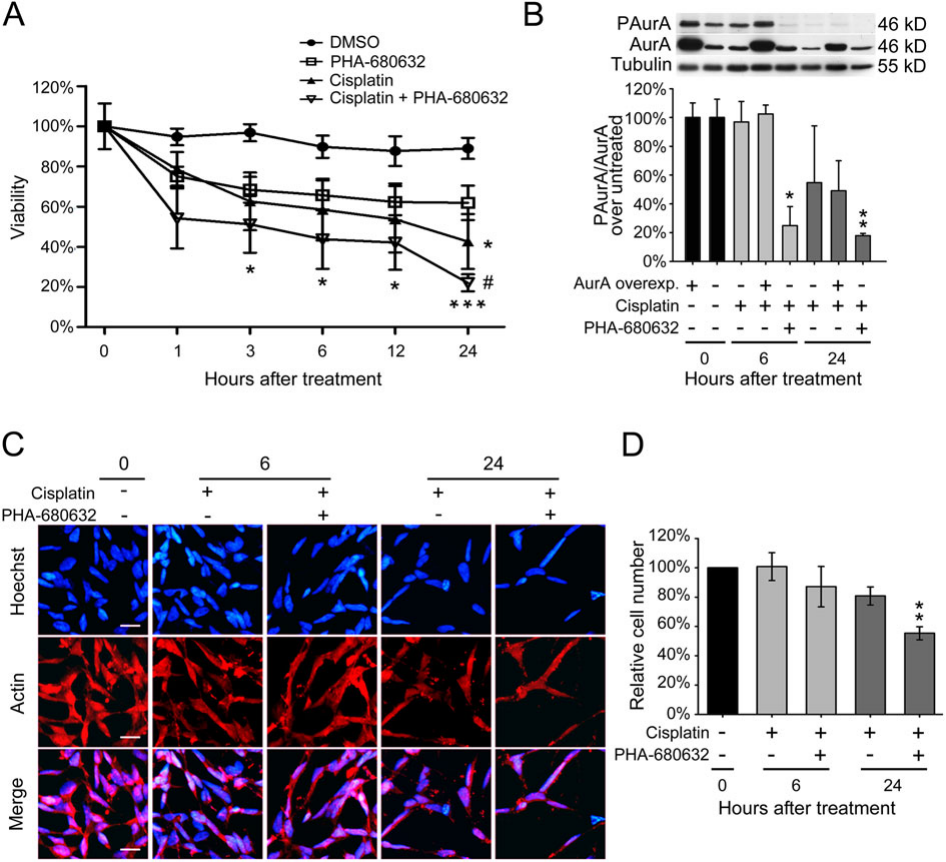
**Combined treatment of cisplatin and PHA-680632 enhances cell mortality.** (A) Residual viability in SK-N-BE cells treated for different hours with cisplatin and/or PHA-680632 (aurora kinase inhibitor). All treatment were performed after 4.5 DIV of differentiation and quantified through colorimetric MTT assay. DMSO treatment represents the control group. Data are given as mean±s.e.m. of percentage viability (*N*=3, each in triplicate). Statistical differences were assessed performing a two-way ANOVA and Bonferroni correction versus control. *P<0.05, ***P<0.001, ^#^*P*=0.012, one-way ANOVA versus aurora kinases inhibitor alone. (B) Densitometric quantification of phospho-aurora in SK-N-BE cells, double normalized on total aurora A protein of each culture, and related to untreated cultures with either normal levels (=100%) or with overexpressed aurora A kinase (=100%). Cells were treated for 6 or 24 h with cisplatin alone or in combination with PHA-680632 inhibitor. Upper panel: representative western blot with tubulin as loading control; lower panel: densitometric analysis from 3 independent experiments. Data are given as percentage mean±s.e.m. *P=0.01, **P=0.006; one-way ANOVA versus untreated control. (C) Immunofluorescence on SK-N-BE cells treated for 6 or 24 h with cisplatin alone or in combination with PHA-680632 inhibitor. Hoechst was used to highlight nuclei and actin was marked to emphasize cell bodies; the merge between the two channels is displayed. (*N*=3; scale bar: 20 µm). (D) Cell count from the immunofluorescence described in panel B. Values are given as mean±s.e.m. of percentage of cell number with respect basal condition (*N*=3). **P=0.003; one-way ANOVA versus untreated control.

We hypothesized that by reducing aurora kinase A activity we may enhance the cytotoxic effect of cisplatin by overcoming the increased production of BDNF in surviving neuroblastoma cells, as a consequence of reduced aurora kinase-dependent BDNF translation. To test this hypothesis, we treated SK-N-BE cells with the aurora kinases inhibitor, PHA-680632 (Soncini et al., 2006), either alone (10 µM) or in combination with cisplatin (5 µg/ml) in serum-free medium for 1, 3, 6, 12 and 24 h. Using a MTT viability assay we found that the aurora kinases inhibitor alone was poorly effective in reducing cell survival with respect to control conditions (i.e. cells treated with vehicle DMSO, set to=100% survival at time zero). Treatment with cisplatin alone caused about 43% survival by 24 h (*P<0.05, two-way ANOVA), while the combined treatment with cisplatin and the aurora kinases inhibitor caused a dramatic reduction in cell viability at all time points tested, starting from 3 h of treatment and reaching 22% of viability after 24 h (Fig. 3A) (*P<0.050, ***P<0.001, two-way ANOVA versus control; ^#^*P*=0.012, one-way ANOVA versus aurora kinases inhibitor alone). In conclusion, a statistically significant decrease in cell viability was present at all tested time points, but at 24 h the reduction was particularly relevant (Fig. 3A). These results also show that there is not a synergic effect of cisplatin+aurora kinase inhibitor, PHA-680632. Fig. 3B shows the effectiveness of aurora kinase inhibitor in reducing aurora A phosphorylation, which was significantly decreased after 6 h of treatment (*P=0.010, *P*=0.006 after 24 h, one-way ANOVA versus untreated control), while PHA-680632 had no effect on total aurora A kinase protein levels. Overexpression of aurora kinase A induced only a slight increase in its phosphorylation which was not significantly affected by 6 and 24 h cisplatin. The effects of cisplatin alone or cisplatin+PHA-680632 combined treatment at 6 and 24 h were also measured by counting the number of cells, identified through Hoechst staining for cell nuclei and actin for cytoplasm. This quantitative assessment of cell number was achieved with a stereological unbiased procedure carried out in three different fields randomly positioned in each well of 24-wells plates (*N*=3 cultures) in the different treatment conditions (Fig. 3C,D). A significant reduction in cell number with respect to the untreated control, which was set as=100%, was observed after 24 h of combined treatment with cisplatin and aurora kinase inhibitor (**P=0.003; one-way ANOVA; Fig. 3D), thus providing a further corroboration of the results obtained with the MTT assay.

### Effects of aurora kinases inhibition on BDNF protein production

**Fig. 4.**
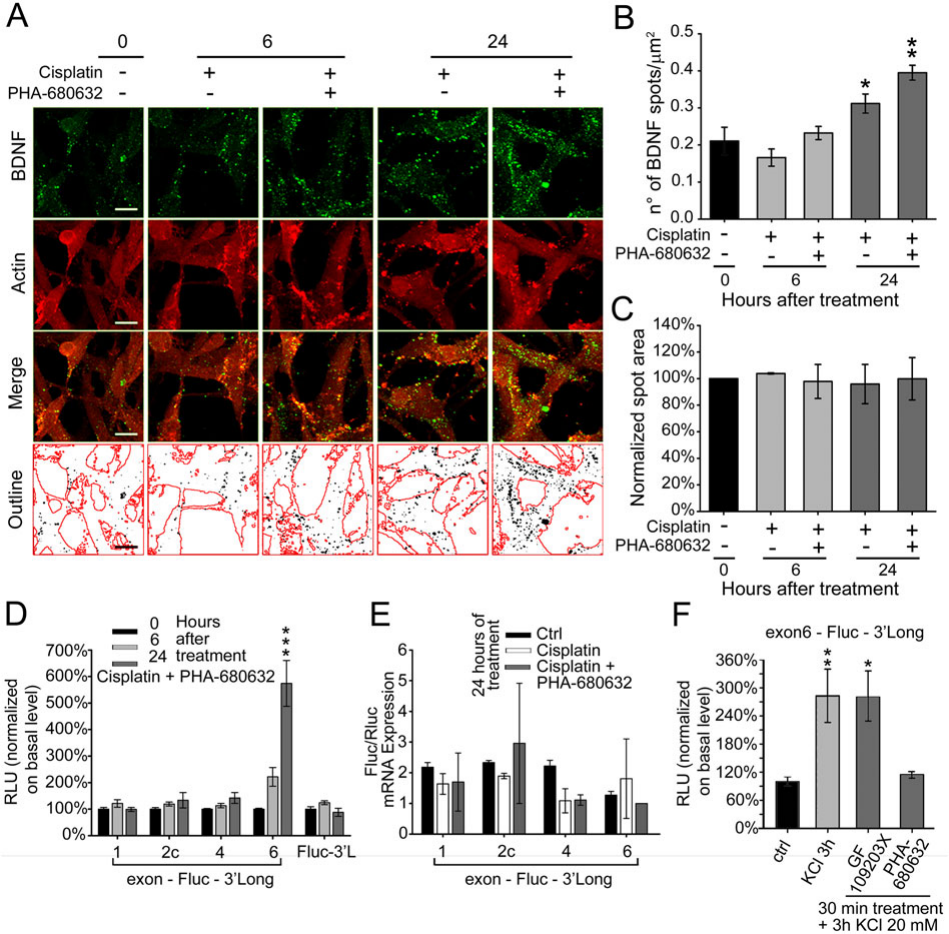
**Increment in BDNF protein production and exon-6 translation upon combined treatment of cisplatin and PHA-680632.** (A) Immunofluorescence on SK-N-BE cells treated with cisplatin±PHA-680632 (aurora inhibitor) at different time points: untreated control (0), 6 and 24 h. BDNF spots are detected using a mouse mAb and actin is marked using a rabbit pAb to emphasize cell bodies; the merge of the two channels is shown as well as its outline, where black dots represent the BDNF spots and red lines the edges of the cell bodies (*N*=3; scale bar: 10 µm). (B) BDNF spot number analysis from the immunofluorescence described in panel C. Data are given as number of spots per µm^2^. *N*=3; *P=0.039, **P=0.004; one-way ANOVA versus untreated) (C) BDNF spot area analysis, normalized to untreated control. (D) Translational capacity of different BDNF isoforms in differentiated SK-N-BE treated as described in panel A. The isoforms tested and the normalization procedures are the same as described in Fig. 2, panel C. RLU, relative luciferase unit. *N*=3, each in duplicate. ***P<0.001; one-way ANOVA untreated control). (E) Quantitative real-time PCR to evaluate the effects of treatment, for 24 h with cisplatin±PHA-680632, on the expression of firefly luciferase (Fluc) mRNA, normalized to the expression of the *Renilla* luciferase used as transfection control (*N*=2). (F) Effect on exon 6-Fluc-3′Long efficiency of translation after 20 mM KCl treatment for 3 h, alone or in combination with GF 109203X (PKC inhibitor) or PHA-680632 (aurora inhibitor) for 30 min of treatment. *N*≥3; *P=0.03, **P=0.006; one-way ANOVA versus control). For all panels, data are given as mean±s.e.m.

Assuming that inhibition of aurora kinases would cause a decrease in BDNF protein levels, we verified the effective inhibition of BDNF protein expression using an anti-BDNF specific monoclonal antibody to visualize the protein by immunofluorescence, and an anti-actin antibody to visualize cell boundaries. BDNF protein was clearly detected as bright fluorescent spots inside neuroblastoma cells (Fig. 4A, BDNF in green, actin in red). Of note, to generate this figure and for better visual comparison, small fields containing a similar number of cells were selected, while the general appearance of the cultures is shown in Fig. 3 and assessment of spot number was achieved with a stereological unbiased procedure carried out in five different fields randomly positioned in each well. In the last row of Fig. 4A, cell boundaries identified on the basis of actin staining are outlined in red and BDNF spots are reported as black dots on a white background, this representation was used to count spots. The count of individual spots normalized for cell area, defined on the basis of actin staining, is shown in Fig. 4B expressed as number of spots per micron square (spots µm^−2^). In particular, under basal conditions we counted a density of 0.21±0.04 BDNF spots µm^−2^ (mean±s.e.m.) which remained unchanged after 6 h of treatment either with cisplatin alone or combined to PHA-680632, rising to 0.31±0.03 BDNF spots µm^−2^ after 24 h of treatment with cisplatin alone (*P*=0.039, one-way ANOVA) and up to 0.40±0.02 after treatment with both cisplatin and the aurora kinase inhibitor (*P*=0.004, one-way ANOVA). Notably, the mean BDNF spot area did not change upon treatments with respect to untreated control (Fig. 4C). In conclusion, this analysis revealed a clear enrichment of BDNF spots in surviving neuroblastoma cells after 24 h of combined treatment with cisplatin and PHA-680632. Thus, the 22% of neuroblastoma cells remaining in the culture after the combined cisplatin+PHA-680632 treatment showed a paradoxical increase in BDNF.

To investigate the contribution of individual transcripts to BDNF increase we used the luciferase translation assay and found that while the cisplatin-dependent increase in translation from exons 1, 2c and 4 was completely suppressed at both 6 and 24 h by combined treatment with PHA-680632, translation from exon 6 showed a strong increase of about sixfold at 24 h (but not 6 h) of treatment (***P<0.001, one-way ANOVA; see Fig. 4D); again, the 3′UTR long sequence alone did not affect translation under cisplatin+PHA-680632 treatment. SYBR-green qRT-PCR experiments showed that cisplatin treatment alone or in combination with PHA-680632 did not significantly affect the levels of firefly luciferase mRNA expressed by the four constructs and normalized by the mRNA levels of *Renilla* luciferase used as transfection control (Fig. 4E). The experiments described above demonstrated that cisplatin-induced translation of *BDNF* exons 1, 2c and 4 requires active aurora kinase, however, the paradoxical increase of *BDNF* exon 6 translation poses the question if aurora kinase in also involved in regulating this transcript or not. Hence, we tested the role of aurora kinase when BDNF translation is induced with a non-cytotoxic stimulus, consisting in a general mild depolarization obtained with high potassium solution (20 mM KCl). Neuroblastoma cells were treated for 3 h with KCl 20 mM alone or in the presence of PHA-680632 aurora-kinases inhibitor, or 50 nM GF 109203X a selective inhibitor of protein kinase C (PKC) which does not regulate BDNF translation in neuroblastoma cells (Heikkila et al., 1993). High KCl induced an almost 300% increase in exon-6-3′UTR long luciferase activity (**P=0.006, one-way ANOVA) which was not affected by the GF 109203X (PKC-inhibitor; *P=0.011, one-way ANOVA) and was completely abolished by PHA-680632 aurora-kinases inhibitor (*P*=0.796 one-way ANOVA; Fig. 4F). Taken together, these results demonstrate that, in response to a physiological stimulus consisting in cell depolarization, *BDNF* exon 6-containing transcripts are translated using a mechanism which requires aurora kinase, but in the presence of a cytotoxic stimulus, translation of these transcripts is driven by an aurora kinase-independent mechanism.

## DISCUSSION

In this study we found that cisplatin-induced cytotoxic stress was able to stimulate BDNF production in the *MYCN*-amplified neuroblastoma cell line SK-N-BE by enhancing both transcription and translation of multiple BDNF mRNAs. We further showed that the combined treatment with cisplatin and the aurora kinases inhibitor PHA-680632 was very effective in reducing cell survival from 43% with cisplatin alone to 22% with the combined treatment. At the same time we observed a paradoxical increase in BDNF production that was accounted for by a strong translational activation of transcripts bearing exon 6 and the 3′UTR long, while translation of *BDNF* transcripts 1, 2C and 4 was suppressed by the combined treatment. Taken together, these results suggest that drug-resistance and cell survival in cisplatin-treated neuroblastoma cells could be mediated by increased BDNF production in response to cytotoxic stress, in particular through enhanced translation of exon-6 with an aurora kinase-independent mechanism.

BDNF is generated as a unique protein precursor encoded by a single coding sequence (CDS), but its gene regulation is highly complex because of the presence of multiple untranslated regions which are alternatively spliced to give rise, in humans, to 34 transcripts, each of which is independently transcribed (Aid et al., 2007; Pruunsild et al., 2007, 2011). It has previously been demonstrated that different *BDNF* mRNA variants target different cell compartments, especially in neurons, in an activity-dependent manner (Tongiorgi et al., 1997); for example, exons 1, 3, 5, 7, 8 and 9a preferentially segregate to soma, exon 4 to proximal dendrites while exons 2 and 6 reach the distal dendrites (Baj et al., 2013, 2011). In addition, they display different ability to be translated in response to various compounds (Vaghi et al., 2014). Concerning the role of BDNF in neuroblastoma tumours, previous studies suggested that BDNF and its receptor TrkB are involved in an autocrine loop that promotes cell survival and resistance to chemotherapy (Baj and Tongiorgi, 2009; Jaboin et al., 2002). Here we demonstrate that 24 h of cisplatin treatment and serum deprivation are able to increase both total BDNF protein and the transcripts encoding all 5′UTR variants, with the exception of exons 5 and 8. Notably, changes in BDNF protein levels do not match mRNA levels, a well-established phenomenon that has been previously described also in normal brain (Tropea et al., 2001). In addition, in this study we show that cisplatin boosted translation of a firefly reporter gene flanked by 5′ and 3′UTRs of *BDNF* transcript (i.e. the 5′UTR exons 1, 2c, 4 or 6 and the 3′UTR long sequence), highlighting the involvement of mechanisms that control the induction of BDNF translation. These findings provide an explanation to previous observations from our lab in which silencing of exons 4 and 6 resulted in a significant reduction in SK-N-BE viability under cisplatin treatment (Baj and Tongiorgi, 2009). In conclusion, one of the major findings of the present study is that cisplatin induces upregulation of BDNF at both transcriptional and translation level.

How can cytotoxic conditions induced by cisplatin promote BDNF translation? Cisplatin [cis-diamminedichloro platinum(II)] is a metal compound commonly used in clinic as an antitumor drug that induces cell damage mainly by cross-linking genomic DNA and, to a lesser extent, proteins. DNA damage triggers different phenomena: on one hand, it causes cell death by necrosis (mainly by PARP-induced ATP depletion) (Fuertes et al., 2003) and/or apoptosis both via intrinsic and extrinsic programmed cell death pathways, leading to final activation of caspase-3-6-7 (Zhou et al., 2002). On the other hand, DNA damage promotes cell cycle arrest, DNA repairing mechanisms and activation of cell survival pathways. These cisplatin-induced pathways require functional activity of transduction mediators, like Akt, ERK and mTOR, which are known also to control protein translation (Cepeda et al., 2007; Cunha et al., 2010). It has previously been shown that BDNF resistance to cisplatin specifically involves rescue from cell-death by TrkB-mediated activation of the PI3K/Akt pathway (Jaboin et al., 2003). Interestingly, BDNF/TrkB signaling was shown to facilitate local translation at synapses by activation of mTOR via PI3K, and both mTOR and ERK are able to regulate the assembly of the elF4E complex, contributing to translation induction (Schratt et al., 2004). These previous findings support the view that cisplatin may directly activate the pathways which control 5′cap-dependent BDNF translation.

Aurora kinase/CPEB is another pathway which is implicated in translation. This mechanism was previously described for CPE-containing α-Ca^2+^/calmodulin-dependent protein kinase II (αCaMKII) mRNA and is highly conserved among vertebrates (Huang et al., 2002). Briefly, upon binding of CPEB and other proteins to the cap-binding subunit (elF4E), mRNAs are kept translationally dormant and are characterized by short poly(A) tails. The phosphorylation of CPEB by aurora kinase stimulates mRNA polyadenilation and the release of elF4E, which is then free to bind and recruit the 40S ribosomal subunit and start scanning the mRNA for the translation initiation (Huang et al., 2002; Pestova et al., 1998). Considering that *BDNF* transcripts bear the CPE element in their 3′UTR, we hypothesised that BDNF translation could use a mechanism similar to that mediated by aurora kinase for αCaMK II mRNAs. Therefore, by blocking aurora kinase activity with the PHA-680632 inhibitor (Soncini et al., 2006), we were expecting increased cell mortality due to a dual effect: altered cell cycle control and decreased BDNF protein translation. While we found that translation of exon 1, 2c and 4 was indeed blocked by the inhibition of aurora kinase, thus confirming the role of this kinase in BDNF translation, exon 6 escaped this blockade. However, when BDNF translation was induced by a non-cytotoxic, physiological stimulus such as membrane depolarization, we found that also exon 6 translation was driven by an aurora kinase-dependent mechanism. Of note, even if the combination of PHA-680632 and cisplatin enhanced exon 6 translation, we observed an increased cell death suggesting a potentially additive chemotherapeutic effect. It was previously shown that cisplatin promotes cell death though activation of an apoptotic pathway in several types of tumours (Bottone et al., 2008). Our study did not investigate if enhanced cell death following combined treatment with cistplatin and PHA-680632 also occurs through apoptosis, and therefore future investigations are required.

It is noteworthy that *BDNF* exon 6 transcript has been reported to be translationally repressed under resting conditions, pointing out a tight regulation of translation for this *BDNF* variant (Vaghi et al., 2014; Baj and Tongiorgi, 2009). Interestingly, it has previously been hypothesized that transcripts bearing exon 6 may contain an internal ribosome entry sites (IRES) sequence which may be activated when 5′ cap-dependent translational mechanisms are repressed (Zaitsev and Lu, 2003). Thus, this *BDNF* transcript which represents also the main transcript present in distal dendrites (Baj et al., 2013) may provide the template for a rapid and massive production of BDNF in conditions of cellular stress able to block 5′ cap-mediated translation (Pinkstaff et al., 2001). It is conceivable that while translation of all other *BDNF* transcripts is efficiently suppressed by the combination of cisplatin and aurora kinase inhibitor, the presence of a putative IRES within the exon 6 may allow this transcript to escape from repression of canonical 5′CAP-dependent translation; however demonstration that an IRES-like mechanism is at the basis of the paradoxical increased translation of *BDNF* exon-6 goes beyond the scope of this study, and therefore further researches are warranted. One further limitation of this study is that while cisplatin behaviour in cultured neuroblastoma cells was shown to be similar to that *in vivo* (Bassili et al., 2010), the combined effects with the aurora kinases inhibitor PHA-680632 should be replicated also *in vivo*, which is a matter for future investigations. Moreover, although the SK-N-BE cell line is a well-accepted model of *MYCN*-amplified aggressive neuroblastoma, experiments on additional cell lines may further validate our findings

In conclusion, the observations made could have important future implications in the use of cisplatin and Aurora kinase inhibitors in combination for the neuroblastoma treatment. Although their combined use show increased activity in the induction of neuroblastoma cell death, specific attention should be paid to the possibility that they could lead to the selection of resistant cells expressing high BDNF levels.

## MATERIALS AND METHODS

### Cell cultures and reagents

Human neuroblastoma cell lines SK-N-BE were grown and differentiated as previously described (Baj and Tongiorgi, 2009). Briefly, cells were cultured in Dulbecco's Modified Eagle's Medium (DMEM) with stable L-Glutamine, 100 U ml^−1^ penicillin, 100 µg/ml streptomycin and 10% decomplemented fetal bovine serum (FBS) (all purchased from EuroClone, Milan, Italy) at 37°C with 5% CO_2_. Differentiation was induced 24 h after plating by adding 5 µM of 9-cis retinoic acid (R4643, Sigma-Aldrich) to cultures and replaced after 48 h. Differentiated cells (4.5 days) were treated with 5 µg ml^−1^ cisplatin with or without 10 µM of the Aurora kinases inhibitor PHA-680632 (both drugs were a gift from P. Carpinelli, Nerviano Medical Sciences) in serum free medium (SFM). GF 109203X (PKC-inhibitor) was purchased from Sigma-Aldrich (B6292) and used at 50 nM (Heikkila et al., 1993).

### Real-time PCRs

For quantification of BDNF cds and isoforms, total RNA was extracted, using TriZol^®^ Reagent (15596026, Invitrogen by Thermo Fisher Scientific, Waltham, MA, USA), from differentiated SK-N-BE cells after different hours of cisplatin treatment: 1, 3, 6, 12 and 24 h and a non-treated control. One microgram of total RNA was reverse-transcribed into cDNA as previously described (Baj and Tongiorgi, 2009) and employed for a quantitative real time PCR (qRT-PCR), performed using the Rotor Gene 6000 instrument according to the manufacturer's instructions. The PCR reactions were carried out in a final volume of 20 µl with 2× Fluocycle II, Master SYBR Green Mix (ERD001250, EuroClone, Milan, Italy), 0.5 µM of each primer and 1 µl of cDNA was added as template. The qRT-PCR was used to evaluate both total and BDNF splice variants expression after cisplatin treatment at the given time points. For quantification of firefly (Fluc) and *Renilla* (Rluc) luciferases mRNA, differentiated SK-N-BE cells were transfected with plasmid vectors as described later in the ‘Luciferase assay’ paragraph. After treatment for 24 h with cisplatin±PHA-680632 inhibitor, total RNA was extracted as described above, treated with DNase I (18068015, Invitrogen by Thermo Fisher Scientific) then 1 µg was reverse-transcribed using SuperScript III (18080044, Invitrogen by Thermo Fisher Scientific). The qRT-PCR reactions were carried out in a final volume of 12 µl with 2× PowerUp™ SYBR^®^ Green Master Mix (A25741, Applied Biosystem, by Thermo Fisher Scientific), 0.2 µM of each primer and 1/20 µl of cDNA was added as template. Rluc expression was used as transfection control. All primer pairs used along with the PCR conditions are listed in Table 1.

**Table 1.**
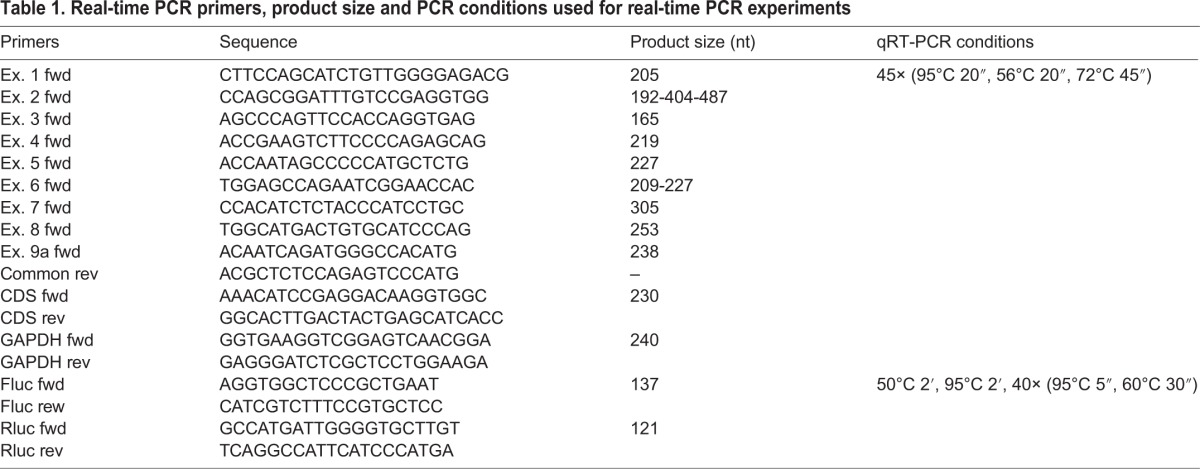
**Real-time PCR primers, product size and PCR conditions used for real-time PCR experiments**

### ELISA assay for total BDNF detection

BDNF levels in differentiated SK-N-BE cells were assessed using the ELISA BDNF Emax Immunoassay System (G7610, Promega Corporation, Madison, WI, USA) at the same time points evaluated in real-time PCR. Briefly, cells in treated and control conditions were lysed and 12.5 µg of total protein were loaded on pre-coated wells according to manufacturer's instructions.

### Western blotting

Differentiated SK-N-BE cells were lysed on ice in lysis RIPA buffer [50 mM Tris-HCl pH 8.0, EDTA 1 mM, 150 mM NaCl, 1% Triton X-100, 0.5% sodium deoxycholate, 0.1% SDS, protease inhibitor cocktail (P8849, Sigma-Aldrich)]. Protein quantification was performed via Bradford assay (ab119216, Abcam, Cambridge, UK), according to manufacturer instructions. Proteins were resolved into 15% SDS-PAGE, loading equal amount of total protein (20 µg) and electroblotted onto nitrocellulose membrane. Membranes were cut above the 35 KDa marker band and differentially processed. The lower portion of the membrane was blocked with fat-free 10% milk in PBS-T [Phosphate Buffer Saline, 0.1% (v/v) Tween-20] and then incubated at 4°C in overnight gentle-shaking with anti-BDNF N-20 antibody (1:500 in blocking solution; sc-546, Santa Cruz, Texas, USA). After three wash steps with PBS-T, the membranes were incubated for 1 h at room temperature with anti-rabbit HRP-conjugated antibody (1:10,000 in fat-free 5% milk in PBS-T; A0545 Sigma-Aldrich). The upper part of the membrane was blocked with fat-free 5% milk in PBS-T, then incubated overnight at 4°C alternatively with the following antibodies: anti-phospho Aurora kinases (1:1000, polyclonal; 2914S, Cell Signaling Technology, MA, USA), anti-aurora kinase A (1:1000, polyclonal; 3092S, Cell Signaling Technology), or with anti-αTubulin antibody (1:8000, monoclonal; T6074, Sigma-Aldrich). After three washing steps, a further incubation for 1 h at room temperature was performed with secondary HRP-conjugated antibodies, specifically anti-rabbit against primary polyclonal antibodies (1:10,000 in fat-free 5% milk in PBS-T), or anti-mouse for monoclonal antibody (1:10,000 in fat-free 5% milk in PBS-T; RABHRP1, Sigma-Aldrich). Thereafter, all the membranes were washed three times and developed with ECL Prime (GE Healthcare, Buckinghamshire, UK) on X-ray film (AU1102, Aurogene S.R.L., Rome, Italy). Densitometric analyses of the western blots were performed using Quantity One software (Bio-Rad Laboratories, CA, USA), according to user manual.

### Luciferase assay

Luciferase assays, to evaluate the translational capacity of BDNF isoforms, were performed as described previously (Vaghi et al., 2014). Briefly, plasmid vectors containing 5′ UTR exons 1, 2c, 4 or 6 and 3′URT long cloned at the edges of a firefly luciferase (Fluc) reporter were used to test the influence of BDNF UTRs on efficiency of translation in stimulated conditions. Around 3000 SK-N-BE cells per well were seeded in 96-well plate designed for luminescence assays (BD Falcon, CA, USA) and induced to differentiate, as described. Differentiated cells were co-transfected with one of the above construct along with a vector containing a *Renilla* (Rluc) reporter as transfection control. Transfection was stopped after 6 h by replacing media or starting the 24 h-point cisplatin treatment with/without aurora kinase inhibitor in SFM. The day after, a 6 h-point treatment was also performed before carrying out the Dual-Luciferase^®^ (E1910, Promega Corporation) assay in an automated Glomax plate reader (Promega Corporation), according to manufaturer's instructions.

### Cell-survival analysis

A colorimetric MTT assay was performed to assess cell survival. 5000 SK-N-BE cells were plated in 96-well plates, maintained, let to differentiate, then treated as described in the ‘Real-Time PCR’ paragraph. Viability was assessed using 3-(4,5-dimethylthiazol-2-yl)-2,5-diphenyltetrazolium bromide (MTT, TOX1, Sigma-Aldrich) following manufacturer's instructions.

### Immunofluorescence and microscopy

Immunofluorescence on SK-N-BE cells was performed as follows, after fixation in 4% paraformaldehyde (PFA) in phosphate-buffer-saline (PBS) for 20 min at room temperature, cells were washed with PBS and permeabilized for 15 min with PBS-Triton X-100 0.1% (PBS-T), then blocked with PBS-T and 2% BSA. Thereafter, cells were incubated for 2 h at room temperature with primary antibodies diluted 1:50 in blocking solution. After three washes in PBS, cells were incubated 1 h at room temperature with secondary antibodies conjugated with Alexa Fluor 488 or 568 fluorophores, diluted 1:200 in PBS-T, then washed with PBS and stained with Hoechst to label nuclei. Finally, coverslips were mounted in Mowiol antifade compound (Sigma-Aldrich). The following antibodies were employed: Mouse monoclonal anti-BDNF (B5050, Sigma-Aldrich); Rabbit polyclonal anti-actin (A5060, Sigma-Aldrich); anti-mouse Alexa Fluor 488 (A11001, Invitrogen); anti-rabbit Alexa Fluor 568 (A10042, Invitrogen).

Images for cell counting were acquired using a Nikon Eclipse E800 epifluorescence microscope with a 20× objective and a Nikon DXM1200 camera. Cells were manually counted with a stereological unbiased procedure and counted using the ‘optical dissector’ method (West, 2002; Mariotti et al., 2002; Tongiorgi et al., 2003), obtained by superimposing a 100×100 µm grid onto 3 different fields of 674×539 µm randomly positioned in each well of 24-wells plate. This grid generated about 30 fields, and according to the stereological principles, only 5 were used to count cells starting by chance from a number from 1-30 and avoiding counting cells in contact with the right and lower boundaries (Tongiorgi et al., 2003; West, 2002). In total, 1/6 of the reference space was sampled. The number of cells is expressed as relative percentage with respect to the untreated condition set as=100% (average from *n*=3 cultures). Images for BDNF expression analysis were acquired using a Nikon Eclipse C1si confocal microscope system mounted on a Nikon TE-2000U inverted microscope with a 60× objective; BDNF spots were counted using ImageJ 1.45b software (NIH, Bethesda, USA) and expressed over the cell area in µm^2^. Briefly, images were processed for *z*-stack projections and thresholds were applied to highlight spots and cells area and then analysed using the Analyze Particle tool. All images from different treatment conditions were processed using the same parameters.

### Statistical analysis

For MTT assay, we performed a two-way ANOVA followed by Bonferroni correction versus DMSO (control) group, using GraphPad Prism, Version 5.03 (GraphPad Software, Inc.), also to generate the graph. All other statistical analysis and graphs were performed using SigmaPlot 11.0 (Systat Software, Inc.). One-way ANOVAs followed by Holm-Sidak corrections against control were performed to compare treated groups against controls. For all analyses, the statistical significance was set at *P*<0.05.
